# Safety profile of tyrosine kinase inhibitors used in non-small-cell lung cancer: An analysis from the Italian pharmacovigilance database

**DOI:** 10.3389/fonc.2022.1005626

**Published:** 2022-11-23

**Authors:** Maria Antonietta Barbieri, Emanuela Elisa Sorbara, Giuseppe Cicala, Vincenza Santoro, Paola Maria Cutroneo, Tindara Franchina, Mariacarmela Santarpia, Nicola Silvestris, Edoardo Spina

**Affiliations:** ^1^ Department of Clinical and Experimental Medicine, University of Messina, Messina, Italy; ^2^ Sicilian Regional Pharmacovigilance Centre, University Hospital of Messina, Messina, Italy; ^3^ Department of Human Pathology in Adulthood and Childhood Gaetano Barresi, University of Messina, Messina, Italy

**Keywords:** non-small cell lung cancer, epidermal growth factor receptor, anaplastic lymphoma kinase, tyrosine kinase inhibitors, adverse drug reactions, pharmacovigilance

## Abstract

**Introduction:**

Non-small cell lung cancer (NSCLC) is often caused by molecular alterations that can be detected by predictive biomarkers including mutations or amplifications of several genes. Several tyrosine kinase inhibitors (TKIs) have been approved in Europe by the European Medicines Agency (EMA) for NSCLC. The aim of this study was to analyze the onset of adverse drug reactions (ADRs) related to TKIs in NSCLC through a spontaneous reporting system (SRS) database.

**Methods:**

All ADR reports having as suspected drug afatinib (AFT), alectinib (ALEC), brigatinib (BRG), ceritinib (CER), crizotinib (CRIZ), erlotinib (ERL), gefitinib (GEF), lorlatinib (LORL), nintedanib (NTB), and osimertinib (OSI) recorded into the Report Reazioni Avverse dei Medicinali (RAM) system database for national data and into the Italian SRS database for Sicilian data and collected from 2006 to 2021 have been evaluated. A descriptive analysis of basal demographic and drug-related characteristics was performed. A case-by-case methodology was conducted paying particular attention to all serious ADR reports collected in Sicily, focusing on type of seriousness, age, sex, concomitant drugs, and comorbidities.

**Results:**

Of the 3,048 Italian reports, most of ADRs were related to ERL (*n* = 1,448), followed by AFT (*n* = 435) and GEF (*n* = 366). ADR reports were slightly more frequent in females (52.2%) and in the age group >65 years (53.0%). A higher number of cases were related to skin disorders (*n* = 1,766; 57.9%), followed by gastrointestinal disorders (*n* = 1,024; 33.6%), general disorders and administration site conditions (n = 536; 17.6%), and infections (*n* = 483; 15.8%). The case-by-case assessment of Sicilian ADRs showed that 33 cases were serious (12.5%) and mainly involved ERL (*n* = 17; 51.5%), occurring in males with a higher onset of respiratory diseases (30.3%) such as respiratory failure, interstitial lung disease and dyspnea.

**Discussion:**

The analysis of spontaneous ADR reports of TKIs confirmed, in general, well-known risks, which often include skin, gastrointestinal, general, liver, and respiratory diseases as well as infections. However, more attention should be paid to the occurrence of serious life-threatening ADRs including respiratory failure, interstitial lung disease, and cardiogenic shock, especially in young patients.

## Introduction

Non-small cell lung cancer (NSCLC) corresponds to about the 85% of lung cancer cases worldwide, that is considered as the second leading cause of cancer and it is responsible for the highest number of deaths (1.8 million deaths, 18% of the total cancer deaths) (1). The incidence is different between males and females (14.3% *vs.* 8.4%) and the primary diagnosis consistently increases over 45 years (2–4). However, the incidence tends to decrease for men, while it is stable or slightly increasing in women considering the smoke habits (1). Symptoms usually appear when the disease is advanced and include cough in 50-75% of patients, hemoptysis, chest pain, dyspnea, sudden weight loss, and bone pain (5). NSCLC patients at stages I, II, and III can be treated surgically or with a radiotherapy approach also in combination with chemotherapy concomitantly or sequentially. Patients with advanced NSCLC are considered unfit for surgery and, in exceptional cases, patients with stage IV and individual metastases in organs, such as brain, adrenal or liver, can be evaluated for surgery but only after pharmacological treatment. For inoperable patients, the first choice is the pharmacological treatment based on histological diagnosis, molecular profile, age, and comorbidities (6).

NSCLC is often caused by molecular alterations that can be detected by predictive biomarkers. In the NSCLC Guidelines, predictive molecular biomarkers include: mutations of the KRAS gene (20-30%), epidermal growth factor receptor (EGFR) mutation (10-15% of Caucasian patients and up to 40% of Asian patients), rearrangements of anaplastic lymphoma kinase (ALK) (3-7%), ROS1 (1-2%), RET (1-2%), NTRK (0.5-1%), mutations of the gene BRAF (2-4%), human epidermal growth factor receptor 2 (HER2) mutation (1-4%), amplification (2-5%) and protein overexpression (2-30%) and amplifications or mutations of the MET gene (2-4%) (7–9). Several tyrosine kinase inhibitors (TKIs) have been approved in Europe by the European Medicines Agency (EMA) for NSCLC: the EGFR inhibitors (EGFRi) afatinib (AFT), erlotinib (ERL), gefitinib (GEF), and osimertinib (OSI); the ALK inhibitors (ALKi) alectinib (ALEC), brigatinib (BRG), ceritinib (CER), crizotinib (CRIZ), and lorlatinib (LORL); the vascular endothelial growth factor (VEGF) inhibitor (VEGFi) nintedanib (NTB). Treatment with TKIs is known to induce adverse events (AE) such as stomatitis and epistaxis with AFT (10), edema and myalgia with ALEC (11), gastrointestinal and liver disorders with CER (12), blood and lymphatic system disorders, vision disturbance and gastrointestinal disorders with CRIZ (13), diarrhea, rash, and paronychia with OSI (14). In addition, ERL increase the incidence of grade 3 or higher AE including rash, diarrhea, proteinuria and increased liver enzymes (15). Given the clinical relevance of some AEs not yet fully characterized it may be useful to use real-word data to identify new and unexpected adverse drug reactions (ADRs).

The aim of this study was 1) to highlight all Italian open data-related ADRs associated with TKIs approved for NSCLC and reported in the Adverse Drug Reactions Report (RAM) system and, consequently, 2) to focus on all regional Sicilian complete reports into the National Pharmacovigilance Network (Rete Nazionale di Farmacovigilanza, RNF) database through a case-by-case evaluation of ADRs.

## Materials and methods

### Data source

A retrospective observational study was conducted in the period January 2006-December 2021 to evaluate the safety of drugs approved for the treatment of NSCLC in a real-world scenario. In Italy, all suspected ADR reports are collected through the RNF. The RNF was established in 2001 by the Italian Medicines Agency (Agenzia Italiana del Farmaco, AIFA) with the aim of collecting all suspected ADRs from drugs and vaccines reported by health professionals and citizens from all Italian regions. According to Italian pharmacovigilance standards, all Italian regions, including Sicily, have full access to their own regional spontaneous reporting data in the RNF and carry out post-marketing surveillance activities through the Pharmacovigilance Regional Centers (PRCs). Since July 2017, AIFA has created a public online access system called RAM monthly updated which allows to check all aggregated data related to national ADR reports registered since 2002 into the RNF. The research in the RAM system can be performed by the trade name or by the active substance indicated as suspected. Reports can be consulted by year, seriousness, age group, gender, while ADRs by the Medical Dictionary for Regulatory Activities (MedDRA^®^) System Organ Class (SOC) and Preferred Term (PT).

In the RNF, PRCs can analyze all detailed regional ADR reports. Each ADR report contains information on the patient, a detailed description of ADR with the relative time to onset (TTO), seriousness, outcome, dechallenge, rechallenge, and relevant laboratory tests, data related to suspected and concomitant drugs with their dosage, frequency, route of administration and therapeutic indication, and all potential comorbidities. Drugs are codified using the Anatomical Therapeutic Chemical (ATC) classification, while suspected ADRs are grouped according to the MedDRA^®^ (16). Suspected ADRs are classified as serious if they were life-threatening or fatal, required hospitalization, led to persistent or significant disability, or a congenital anomaly/birth defect or have been defined as other medically important conditions (17). The TTO was expressed in days and was calculated as the time elapsed between the start of drug use and the beginning of the ADR.

### Case definition and selection criteria

All national aggregated ADR reports collected into the RAM system from the date of drug approval until December 31, 2021 and related to the following TKIs approved for NSCLC were consulted: AFT, ALEC, BRG, CER, CRIZ, ERL, GEF, LORL, NTB, and OSI. Moreover, only cases related to the branded name “Vargatef^®^” for NTB were included to avoid some therapeutic bias with other available medications having NTB as active substance and approved for other indications. Entrectinib and selpercatinib were excluded from the study due to their recent approval for the treatment of NSCLC, while larotrectinib was not included because it is not yet approved in Italy.

Furthermore, all Sicilian detailed ADR reports collected into the RNF database and having as suspected drug one of the above mentioned TKIs and signaled from January 2006 to December 31, 2021 were collected. Before to proceed with the analysis, literature data and duplicates were excluded from the database.

### Statistical analysis

A descriptive analysis of publicly available aggregated national data coming from the RAM system and of regional full access detailed reports was performed to assess demographic characteristics and drug-related variables. Specifically, analyses for year, sex, age, and seriousness were conducted. All ADRs were analyzed according to MedDRA^®^ classification levels for SOC and PT by grouping each ADR into the corresponding SOC and synonymous PTs of the same clinical condition under a unique term (**Supplementary Table S1**). Absolute and relative frequencies were calculated for all variables.

Consequently, a case-by-case analysis of all detailed reports registered in Sicily was carried out, considering any concomitant drugs, potential comorbidities, outcomes, TTO and the causality assessment. The causal relationship between the ADR and the suspected drug was estimated using the Naranjo’s algorithm, classifying ADRs in highly probable (score ≥ 9), probable (score 5 - 8), possible (score 1 - 4), or doubtful (score ≤ 0) (18).

## Results

### National data

**Figure 1:**
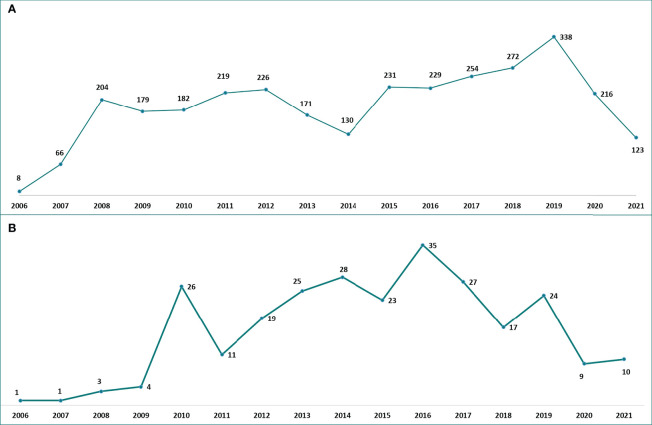
Italian **(A)** and Sicilian **(B)** trend of ADR reports related to TKIs approved for NSCLC over the years. ADR, adverse drug reaction; NSCLC, non-small cell lung cancer; TKIs, tyrosine kinase inhibitors.

This study included a total of 3,048 ADR reports collected from January 1, 2006 to December 31, 2021 into the RAM system and having as suspected drug one of TKIs used for the treatment of NSCLC. A gradual increase of reports was noted over the years with a decrease in 2014 (*n* = 130; 4.3%) and a peak in 2019 (*n* = 338; 11.1%) (**Figure 1**). The trend showed a higher number of reports related to EGFRi: from 2006 to 2016 to ERL (*n* = 1,352; 44.5%), and from 2017 to 2018 to AFT (*n* = 151; 5.0%), while in the last 3 years most of reports concerned OSI (*n* = 240; 7.9%). Focusing on seriousness, the majority of ADRs were not serious (not serious, 71.9% *vs*. serious, 26.7%) and the highest number of serious ADRs (SADRs) was recorded for NTB (*n* = 27; 73.0%), OSI (*n* = 156; 56.5%), and for the ALKi LORL (*n* = 6; 60.0%) and ALEC (*n* = 76; 54.3%). Considering patients’ characteristics, ADR reports were slightly more frequent in females (females, 52.2% *vs.* males, 46.6%) although a higher proportion of males was reported for NTB (*n* = 24; 64.9%), ERL (*n* = 893; 61.7%), and LORL (*n* = 6; 60.0%); the age group >65 years was mainly reported (*n* = 1,617; 53.0%), but the age group 18-65 years was the most shown for ALKi: ALEC (*n* = 82; 58.6%), BRG (*n* = 7; 70.0%), CER (*n* = 32; 61.5%), CRIZ (*n* = 168; 61.3%), and LORL (*n* = 20; 54.1) (**Table 1**).

**Table 1:** Descriptions of Italian ADR reports related to TKIs for NSCLC collected into the RAM system from January 2006 to December 2021.

Suspected drug	Total	Seriousness			Gender			Age group			
		Not serious (%)	Serious (%)	NA (%)	Male (%)	Female (%)	NA (%)	<18 yrs (%)	18-65 yrs (%)	>65 yrs (%)	NA (%)
**EGFRi**	**2,525**	**1,936 (76.7)**	**563 (22.3)**	**26 (1.0)**	**1,206 (47.8)**	**1,299 (51.4)**	**20 (0.8)**	**7 (0.3)**	**914 (36.2)**	**1,463 (57.9)**	**141 (5.6)**
AFT	435	326 (74.9)	104 (23.9)	5 (1.1)	126 (29.0)	301 (69.2)	8 (1.8)		193 (44.4)	211 (48.5)	31 (7.1)
ERL	1,448	1,241 (85.7)	192 (13.3)	15 (1.0)	893 (61.7)	550 (38.0)	5 (0.3)	7 (0.5)	494 (34.1)	929 (64.2)	18 (1.2)
GEF	366	249 (68.0)	111 (30.3)	6 (1.6)	97 (26.5)	267 (73.0)	2 (0.5)		130 (35.5)	214 (58.5)	22 (6.0)
OSI	276	120 (43.5)	156 (56.5)		90 (32.6)	181 (65.6)	5 (1.8)		97 (35.1)	109 (39.5)	70 (25.4)
**ALKi**	**486**	**246 (50.6)**	**224 (46.1)**	**16 (3.3)**	**191 (39.3)**	**280 (57.6)**	**15 (3.1)**		**294 (60.5)**	**142 (29.2)**	**50 (10.3)**
ALEC	140	64 (45.7)	76 (54.3)		59 (42.1)	78 (55.7)	3 (2.1)		82 (58.6)	36 (25.7)	22 (15.7)
BRG	10	8 (80.0)	2 (20.0)		3 (30.0)	7 (70.0)			7 (70.0)	2 (20.0)	1 (10.0)
CER	52	30 (57.7)	19 (36.5)	3 (5.8)	19 (36.5)	31 (59.6)	2 (3.8)		32 (61.5)	16 (30.8)	4 (7.7)
CRIZ	274	140 (51.1)	121 (44.2)	13 (4.7)	104 (38.0)	160 (58.4)	10 (3.6)		168 (61.3)	84 (30.7)	22 (8.0)
LORL	10	4 (40.0)	6 (60.0)		6 (60.0)	4 (40.0)			5 (50.0)	4 (40.0)	1 (10.0)
**VEGFi**											
NTB	37	10 (27.0)	27 (73.0)		24 (64.9)	13 (35.1)			20 (54.1)	12 (32.4)	5 (13.5)
**Total**	**3,048**	**2,192 (71.9)**	**814 (26.7)**	**42 (1.4)**	**1,421 (46.6)**	**1,592 (52.2)**	**35 (1.2)**	**7 (0.3)**	**1,228 (40.3)**	**1,617 (53.0)**	**196 (6.4)**

Bold type intended as drug classes and total number of reports. ADR, adverse drug reaction; AFT, afatinib; ALEC, alectinib; ALKi, anaplastic lymphoma kinase inhibitors; BRG, brigatinib; CER, ceritinib; CRIZ, crizotinib; EGFRi, epidermal growth factor receptor inhibitors; ERL, erlotinib; GEF, gefitinib; LORL, lorlatinib; NSCLC, non-small cell lung cancer; NTB, nintedanib; OSI, osimertinib; TKIs, tyrosine kinase inhibitors; VEGFi, vascular endothelial growth factor inhibitor; NA, not available; RAM, Reazioni Avverse dei Medicinali; yrs, years.

Considering ADRs, they were mostly related to skin and subcutaneous tissue disorders (*n* = 1,766; 57.9%), gastrointestinal disorders (*n* = 1,024; 33.6%), general disorders and administration site conditions (*n* = 536; 17.6%), infections and infestations (*n* = 483; 15.8%), hepatobiliary disorders (*n* = 419; 13.7%), and respiratory, thoracic and mediastinal disorders (*n* = 309; 10.1%). Skin and subcutaneous tissue disorders, especially rash, were mostly reported for EGFRi ERL (*n* = 1,120; 77.3%), AFT (*n* = 277; 63.7%), and GEF (*n* = 214; 58.5%). The onset of gastrointestinal disorders, especially diarrhea, were mainly reported for NTB and AFT (*n* = 31; 83.8% and *n* = 268; 61.6%, respectively). Furthermore, general disorders and administration site conditions were particularly shown for NTB (*n* = 20; 54.1%), LORL (*n* = 4; 40.0%), and ALEC (*n* = 48; 34.3%). Concerning infections, including folliculitis, the EGFRi ERL and AFT had a higher number of related ADRs (*n* = 296; 20.4% and *n* = 79; 18.2%, respectively). The onset of hepatobiliary disorders, mainly hypertransaminasemia, was detected for ALKi CER (*n* = 31; 59.6%), ALEC (*n* = 57; 40.7%), and CRIZ (*n* = 98; 35.8%), but also for NTB (*n* = 12; 32.4%) and GEF (*n* = 111; 30.3%). More than half of ADRs related to blood and lymphatic system disorders were associated with the VEGFi NTB (*n* = 19; 51.4%) (**Table 2**).

**Table 2:** Descriptions of main ADRs associated with TKIs for NSCLC treatment and reported into the RAM system.

Adverse Drug Reaction, *n* (%)	EGFRi				ALKi					VEGFi	Total (*n* = 3,048)
	AFT (*n* = 435)	ERL (*n* = 1,448)	GEF (*n* = 366)	OSI (*n* = 276)	ALEC (*n* = 140)	BRG (*n* = 10)	CER (*n* = 52)	CRIZ (*n* = 274)	LORL (*n* =10)	NTB (*n* = 37)	
**Skin and subcutaneous tissue disorders**	**277 (63.7)**	**1,120 (77.3)**	**214 (58.5)**	**99 (35.9)**	**25 (17.9)**	**5 (50.0)**	**2 (3.0)**	**20 (7.3)**		**4 (10.8)**	**1,766**
Rash	132 (30.3)	605 (41.8)	103 (28.1)	35 (12.7)	17 (12.1)	2 (20.0)	1 (1.9)	6 (2.2)			901
Dermatitis	22 (5.1)	133 (9.2)	20 (5.5)	2 (0.7)		1 (10.0)					178
Pruritus	19 (4.4)	99 (6.8)	24 (6.6)	16 (5.8)	2 (1.4)			3 (1.1)			163
Skin toxicity	38 (8.7)	73 (5.0)	12 (3.3)	9 (3.3)		1 (10.0)		2 (0.7)			135
Nail and nail bed conditions	50 (11.5)	38 (2.6)	17 (4.6)	18 (6.5)				1 (0.4)		2 (5.4)	126
**Gastrointestinal disorders**	**268 (61.6)**	**378 (26.1)**	**101 (27.6)**	**77 (27.9)**	**32 (22.9)**	**2 (20.0)**	**38 (73.1)**	**97 (35.4)**		**31 (83.8)**	**1,024**
Diarrhea	175 (40.2)	214 (14.8)	46 (12.6)	42 (15.2)	3 (2.1)		5 (9.6)	12 (4.4)		16 (43.2)	513
Nausea	16 (3.7)	36 (2.5)	11 (3.0)	7 (2.5)	3 (2.1)	2 (20.0)	7 (13.5)	24 (8.8)			106
Vomiting	15 (3.4)	25 (1.7)	8 (2.2)	3 (1.1)	4 (2.9)		11 (21.2)	20 (7.3)		1 (2.7)	87
Abdominal pain	5 (1.1)	20 (1.4)	8 (2.2)	4 (1.4)	2 (1.4)		7 (13.5)	12 (4.4)		1 (2.7)	59
Stomatitis	22 (5.1)	26 (1.8)	4 (1.1)	4 (1.4)	1 (0.7)			1 (0.4)			58
**General disorders and administration site conditions**	**89 (20.5)**	**202 (14.0)**	**42 (11.5)**	**47 (17.0)**	**48 (34.3)**	**3 (30.0)**	**14 (26.9)**	**67 (24.5)**	**4 (40.0)**	**20 (54.1)**	**536**
Asthenia	15 (3.4)	85 (5.9)	15 (4.1)	14 (5.1)	18 (12.9)		6 (11.5)	12 (4.4)		5 (13.5)	170
Mucosal inflammation	33 (7.6)	30 (2.1)	3 (0.8)	4 (1.4)				1 (0.4)			71
Oedema peripheral	2 (0.5)	3 (0.2)	1 (0.3)		9 (6.4)	2 (20.0)	2 (3.8)	28 (10.2)	2 (20.0)		49
Pyrexia	6 (1.4)	13 (0.9)	3 (0.8)	3 (1.1)	2 (1.4)		3 (5.8)	7 (2.6)			37
Xerosis	3 (0.7)	20 (1.4)	1 (0.3)								24
**Infections and infestations**	**79 (18.2)**	**296 (20.4)**	**49 (13.4)**	**34 (12.3)**	**10 (7.1)**		**1 (1.9)**	**13 (4.7)**		**1 (2.7)**	**483**
Folliculitis	23 (5.3)	136 (9.4)	16 (4.4)	3 (1.1)							178
Conjunctivitis	18 (4.1)	72 (5.0)	6 (1.6)	6 (2.2)							102
Rash pustular	5 (1.1)	18 (1.2)	3 (0.8)	1 (0.4)							27
**Hepatobiliary disorders**	**15 (3.4)**	**63 (4.4)**	**111 (30.3)**	**32 (11.6)**	**57 (40.7)**		**31 (59.6)**	**98 (35.8)**		**12 (32.4)**	**419**
Hypertransaminasaemia	5 (1.1)	14 (1.0)	78 (21.3)	14 (5.1)	20 (14.3)		10 (19.2)	58 (21.2)		10 (27.0)	209
Liver injury	3 (0.7)	1 (0.1)	15 (4.1)	5 (1.8)	6 (4.3)		5 (9.6)	15 (5.5)			50
Hyperbilirubinaemia		18 (1.2)	4 (1.1)	1 (0.4)	18 (12.9)		2 (3.8)	1 (0.4)			44
**Respiratory, thoracic and mediastinal disorders**	**33 (7.6)**	**92 (6.4)**	**44 (12.0)**	**53 (19.2)**	**28 (20.0)**	**1 (10.0)**	**6 (11.5)**	**40 (14.6)**	**3 (30.0)**	**9 (24.3)**	**309**
Dyspnoea	6 (1.4)	35 (2.4)	8 (2.2)	10 (3.6)	8 (5.7)		1 (1.9)	6 (2.2)			74
Interstitial lung disease	7 (1.6)	7 (0.5)	8 (2.2)	13 (4.7)	3 (2.1)	1 (10.0)		14 (5.1)	1 (10.0)		54
Respiratory failure	4 (0.9)	8 (0.6)	7 (1.9)	8 (2.9)	3 (2.1)			5 (1.8)		2 (5.4)	37
**Blood and lymphatic system disorders**	**4 (0.9)**	**32 (2.2)**	**21 (5.7)**	**46 (16.7)**	**18 (12.9)**			**20 (7.3)**		**19 (51.4)**	**160**
Neutropenia		5 (0.3)	5 (1.4)	8 (2.9)	5 (3.6)			23 (8.4)		7 (18.9)	53
Thrombocytopenia		3 (0.2)	6 (1.6)	28 (10.1)	1 (0.7)		1 (1.9)				39
Anaemia	1 (0.2)	16 (1.1)	6 (1.6)	5 (1.8)	6 (2.9)			1 (0.4)		1 (2.7)	36
**Metabolism and nutrition disorders**	**27 (6.2)**	**65 (4.5)**	**14 (3.8)**	**10 (3.6)**	**12 (8.6)**		**11 (21.2)**	**17 (6.2)**	**2 (20.0)**		**158**
Decreased appetite	12 (2.8)	46 (3.2)	6 (1.6)	7 (2.5)			6 (11.5)	6 (2.2)			83
Abnormal loss of weight	2 (0.5)	16 (1.1)	7 (1.9)	4 (1.4)	1 (0.7)		2 (3.8)			1 (2.7)	33
Hypercreatininaemia	3 (0.7)	8 (0.6)	3 (0.8)	2 (0.7)	2 (1.4)		5 (9.6)	5 (1.8)			28
**Nervous system disorders**	**9 (2.1)**	**41 (2.8)**	**34 (9.3)**	**15 (5.4)**	**5 (3.6)**		**3 (5.8)**	**22 (8.0)**	**5 (50.0)**	**10 (27.0)**	**144**
Paresthesia	1 (0.2)	5 (0.3)	2 (0.5)	3 (1.1)						2 (5.4)	13
Dysgeusia	1 (0.2)	3 (0.2)	1 (0.3)					3 (1.1)			8
**Neoplasm bening, malignant and unspecified**	**17 (3.9)**	**8 (0.6)**	**20 (5.5)**	**47 (17.0)**	**1 (0.7)**	**1 (10.0)**	**1 (1.9)**	**15 (5.5)**		**13 (35.1)**	**123**
Neoplasm progression	15 (3.4)	2 (0.1)	12 (3.3)	37 (13.4)	4 (2.9)	1 (10.0)	1 (1.9)	14 (5.1)		17 (45.9)	103
**Eye disorders**	**19 (4.4)**	**59 (4.1)**	**7 (1.9)**	**5 (1.8)**	**1 (0.7)**			**22(8.0)**			**113**
Visual impairment	1 (0.2)	4 (0.3)	1 (03)					7 (2.6)			13
Blepharitis	3 (0.7)	6 (0.4)	1 (0.3)	1 (0.4)							11
Ocular hyperaemia	4 (0.9)	3 (0.2)	2 (0.5)	1 (0.4)				1 (0.4)			11
**Cardiac disorders**	**1 (0.2)**	**14 (1.0)**	**4 (1.1)**	**27 (9.8)**	**7 (5.0)**		**6 (11.5)**	**27 (9.9)**		**3 (8.1)**	**89**
Bradycardia					1 (0.7)		4 (7.7)	18 (6.6)			23
Cardiac failure				9 (3.3)				4 (1.5)		2 (5.4)	15
Cardiac fibrillation	1 (0.2)	1 (0.1)		5 (1.8)	2 (1.4)						9
**Investigations**	**2 (0.5)**	**9 (0.6)**	**17 (4.6)**	**17 (6.2)**	**13 (9.3)**	**1 (10.0)**	**14 (26.9)**	**10 (3.6)**	**3 (30.0)**		**86**
Electrocardiogram QT prolonged				7 (2.5)			5 (9.6)	2 (0.7)			14
Gamma-glutamyltransferase increased		1 (0.1)	3 (0.8)	1 (0.4)			1 (1.9)	4 (1.5)			10
**Musculoskeletal and connective tissue disorders**	**11 (2.5)**	**16 (1.1)**	**9 (2.5)**	**7 (2.5)**	**14 (10.0)**		**2 (3.8)**	**3 (1.1)**	**1 (10.0)**		**63**
Myalgia	4 (0.9)	1 (0.1)		2 (0.7)	7 (5.0)				1 (10.0)		15
Muscle spasms	3 (0.7)	2 (0.1)	3 (0.8)	3 (1.1)	1 (0.7)						12
**Vascular disorders**	**3 (0.7)**	**19 (1.3)**	**8 (2.2)**	**16 (5.8)**	**1 (0.7)**	**1 (10.0)**		**8 (2.9)**		**2 (5.4)**	**58**
Hypotension	1 (0.2)	5 (0.3)	1 (0.3)	1 (0.4)				5 (1.8)			13
Deep vein thrombosis		2 (0.1)		2 (0.7)	1 (12.5)			1 (0.4)		2 (5.4)	8
Flushing	2 (0.5)	4 (0.3)		2 (0.7)							8
**Renal and urinary disorders**	**10 (2.3)**	**10 (0.7)**	**8 (2.2)**	**4 (1.4)**	**5 (3.6)**			**10 (3.6)**	**1 (10.0)**	**1 (2.7)**	**49**
Renal failure	9 (2.1)	4 (0.3)	1 (0.3)	3 (1.1)	3 (2.1)			2 (0.7)			22
Renal cyst								7 (2.6)			7
**Injury, poisoning and procedural complications**	**13 (3.0)**	**7 (0.5)**	**11 (30.)**	**4 (1.4)**	**3 (2.1)**	**1 (10.0)**		**2 (0.7)**			**41**
Off label use	3 (0.7)		4 (1.1)	1 (0.4)		1 (10.0)					9

Bold type intended as System Organ Classes. ADR, adverse drug reaction; AFT, afatinib; ALEC, alectinib; ALKi, anaplastic lymphoma kinase inhibitors; BRG, brigatinib; CER, ceritinib; CRIZ, crizotinib; EGFRi, epidermal growth factor receptor inhibitors; ERL, erlotinib; GEF, gefitinib; LORL, lorlatinib; NSCLC, non-small cell lung cancer; NTB, nintedanib; OSI, osimertinib; TKIs, tyrosine kinase inhibitors; VEGFi, vascular endothelial growth factor inhibitor.

### Regional data

Considering regional data, 263 Sicilian reports related to the use of TKIs for the treatment of NSCLC were collected. On the contrary to Italian reports, the regional ADR reporting rate has not gradually increased over the years: after a considerable peak in 2010 (*n* = 26; 9.9%) followed by a decrease in 2011 (*n* = 11; 4.2%), other peaks were noted in 2016 (*n* = 35; 13.3%) and 2019 (*n* = 24; 9.1%) with a gradual decrease in the last two years. The trend was similar for the higher number of EGFRi-related reports: from 2006 to 2017 most of the reports concerned ERL (*n* = 132; 50.2%), from 2018 to 2020 AFT (*n* =22; 8.4%), while in the last year OSI (n =5; 1.9%) (**Figure 1**). Reports having as suspected drug BRG or LORL were not detected in the Sicilian database. As reported in national data, not serious ADRs occurred more frequently (not serious, 73.0% *vs.* serious, 25.9%). Focusing on gender, a higher number of reports was noted for females except for the EGFRi AFT and GEF that were mostly reported in males (*n* = 29; 69.0% and *n* = 33; 71.7%, respectively). Considering all ADR reports, the age group >65 years was the most described except for ALEC, CER, CRIZ, NTB, and OSI that were mainly associated with the age group 18-65 years (**Table 3**).

**Table 3:** Descriptions of Sicilian ADR reports related to TKIs for NSCLC collected into the RNF from January 2006 to December 2021.

Suspected drug	Total	Seriousness			Gender			Age group		
		Not serious (%)	Serious (%)	NA (%)	Male (%)	Female (%)	NA (%)	18-65 yrs (%)	>65 yrs (%)	NA (%)
**EGFRi**	**238**	**177 (74.4)**	**59 (24.8)**	**2 (0.8)**	**117 (49.2)**	**119 (50.0)**	**2 (0.8)**	**86 (36.1)**	**146 (61.4)**	**6 (2.5)**
AFT	42	25 (59.5)	15 (35.7)	2 (4.8)	29 (69.0)	13 (31.0)		13 (31.0)	27 (64.3)	2 (4.8)
ERL	138	110 (79.7)	28 (20.3)		49 (35.5)	88 (63.8)	1 (0.7)	45 (32.6)	91 (65.9)	2 (1.4)
GEF	46	36 (78.3)	10 (21.7)		33 (71.7)	12 (26.1)	1 (2.2)	20 (43.5)	25 (54.3)	1 (2.2)
OSI	12	6 (50.0)	6 (50.0)		6 (50.0)	6 (50.0)		8 (66.7)	3 (25.0)	1 (8.3)
**ALKi**	**20**	**11 (55.0)**	**8 (40.0)**	**1 (5.0)**	**5 (25.0)**	**15 (75.0)**		**13 (65.0)**	**5 (25.0)**	**2 (10.0)**
ALEC	1	1 (100)				1 (100)		1 (100)		
CER	3	2 (66.7)	1 (33.3)		1 (33.3)	2 (66.7)		3 (100)		
CRIZ	16	8 (50.0)	7 (43.8)	1 (6.3)	4 (25.0)	12 (75.0)		9 (56.3)	5 (31.3)	2 (12.5)
**VEGFi**										
NTB	5	4 (80.0)	1 (20.0)		1 (20.0)	4 (80.0)		4 (80.0)	1 (20.0)	
**Total**	**263**	**192 (73.0)**	**68 (25.9)**	**3 (1.1)**	**123 (46.8)**	**138 (52.5)**	**2 (0.8)**	**103 (39.2)**	**152 (57.8)**	**8 (3.0)**

Bold type intended as drug classes and total numebr of reports. ADR, adverse drug reaction; AFT, afatinib; ALEC, alectinib; ALKi, anaplastic lymphoma kinase inhibitors; CER, ceritinib; CRIZ, crizotinib; EGFRi, epidermal growth factor receptor inhibitors; ERL, erlotinib; GEF, gefitinib; NSCLC, non-small cell lung cancer; NTB, nintedanib; OSI, osimertinib; TKIs, tyrosine kinase inhibitors; VEGFi, vascular endothelial growth factor inhibitor; NA, not available; RNF, Rete Nazionale di Farmacovigilanza; yrs, years.

**Figure 2:**
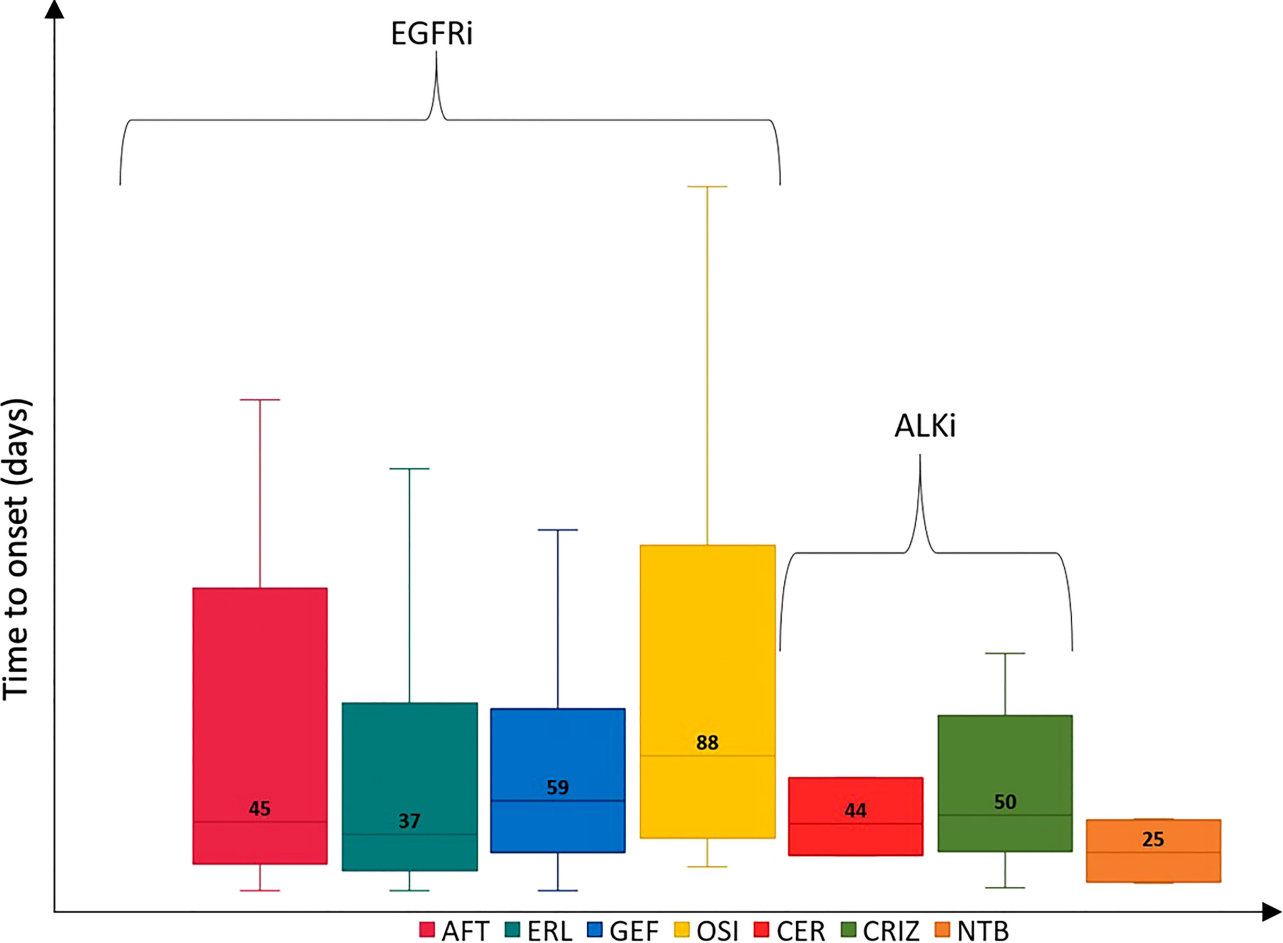
Time to onset of Sicilian ADRs related to TKIs approved for NSCLC. ADR, adverse drug reaction; AFT, afatinib; ALEC, alectinib; ALKi, anaplastic lymphoma kinase inhibitors; BRG, brigatinib; CER, ceritinib; CRIZ, crizotinib; EGFRi, epidermal growth factor receptor inhibitors; ERL, erlotinib; GEF, gefitinib; LORL, lorlatinib; NSCLC, non-small cell lung cancer; NTB, nintedanib; OSI, osimertinib; TKIs, tyrosine kinase inhibitors.

Sicilian TKI-related ADRs were similar to national reports, although other relevant ADRs were the onset of xerosis and decreased appetite with ERL (*n* = 12; 8.7% and *n* = 6; 4.3%, respectively) and the onset of bradycardia with CER (*n* = 2; 66.7%) (**Table 4**). The case-by-case assessment of serious Sicilian ADR reports is described in **Table 5**. Considering all reports with only one TKI reported as suspected, the median TTO of ADRs was almost equal for all EGFRi and ALKi (from 37 to 88 days) but it was slightly lower for NTB which was related to a median (Q1-Q3) TTO of 25 (6.5-44) days (**Figure 2**). Out of the 263 collected reports, SADRs that led to hospitalization, life-threatening, persistent or significant disability, and death were 33 (12.5%) with a median TTO of 57.5 (32.3-135.5) days and mainly involved ERL (*n* = 17; 51.5%), followed by cases related to GEF (*n* = 7; 21.2%), OSI (*n* = 3; 9.1%), CRIZ (*n* =2; 6.1%), and AFT (*n* =1; 3%). Only 2 SADR reports had as suspected drugs a combination of a TKI and other agents approved for NSCLC. The majority of reports involved hospitalization (*n* = 24; 72.7%), followed by death (*n* = 5; 15.2%), life-threatening events (*n* = 3; 9.1%), and persistent or significant disabilities (*n* = 1; 3%). The causality assessment showed that ADRs were mainly possible (60.6%). SADR reports occurred mostly in males (males, 63.6% *vs.* females, 36.4%) and the median age was of 64 (57-69) years. Reports mostly described a higher percentage of respiratory disorders (*n* = 10; 30.3%), including respiratory failure, interstitial lung disease, and dyspnea that occurred in men and were mainly related to ERL (*n* = 9; 90.0%). Four cases of death related to ERL were associated with the onset of epilepsy and cerebral ischemia in a man dead after 49 days, the occurrence of pneumonia with neutropenia and leukopenia in a man dead after 26 days, the onset of respiratory failure and cardiogenic shock in a man dead after 7 days, and the occurrence of intestinal lung disease in a man of 37 years that died after 8 days. Two cases of death were associated with OSI and concerned a case of neoplasm progression in a women of 55 years and a case of sudden death in a man of 48 years.

**Table 4:** Descriptions of main Sicilian ADRs associated with TKIs for NSCLC treatment and reported into the RNF.

Adverse Drug Reaction, *n* (%)	EGFRi				ALKi			VEGFi	Total (*n* = 263)
	AFT (*n* = 42)	ERL (*n* = 138)	GEF (*n* = 46)	OSI (*n* = 12)	ALEC (*n* = 1)	CER (*n* = 3)	CRIZ (*n* = 16)	NTB (*n* = 5)	
**Skin and subcutaneous tissue disorders**	**25 (59.5)**	**86 (62.3)**	**15 (32.6)**	**4 (33.3)**			**2 (12.5)**		**132**
Rash	15 (35.7)	42 (30.4)	9 (19.6)	4 (33.3)			1 (6.3)		71
Pruritus	3 (7.1)	13 (9.4)	3 (6.5)						19
Nail and nail bed conditions	4 (9.5)	7 (5.1)	6 (13.0)	1 (8.3)					18
**Gastrointestinal disorders**	**24 (57.1)**	**25 (18.1)**	**12 (26.1)**	**4 (33.3)**		**1 (33.3)**	**2 (12.5)**	**4 (80.0)**	**72**
Diarrhea	20 (47.6)	19 (13.8)	11 (23.9)	4 (33.3)				3 (60.0)	57
Vomiting	1 (2.4)	3 (2.2)	1 (2.2)				1 (6.3)	1 (20.0)	7
Abdominal pain		2 (1.4)	1 (2.2)	1 (8.3)		1 (33.3)	1 (6.3)		6
**General disorders and administration site conditions**	**6 (14.3)**	**32 (23.2)**	**5 (10.9)**	**3 (25.0)**			**4 (25.0)**	**1 (20.0)**	**51**
Asthenia	1 (2.4)	12 (8.7)	4 (8.7)	1 (8.3)			2 (12.5)	1 (20.0)	21
Xerosis		12 (8.7)							12
Mucosal inflammation	3 (7.1)	2 (1.4)							5
**Infections and infestations**	**3 (7.1)**	**22 (15.9)**	**3 (6.5)**						**28**
Conjunctivitis	3 (7.1)	11 (8.0)	1 (2.2)						15
Folliculitis		12 (8.7)	1 (2.2)						13
**Hepatobiliary disorders**	**1 (2.4)**	**03 (2.2)**	**15 (32.6)**			**1 (33.3)**	**8 (50.0)**	**1 (20.0)**	**29**
Hypertransaminasaemia	1 (2.4)	1 (0.7)	14 (30.4)			1 (33.3)	8 (50.0)	1 (20.0)	26
**Respiratory, thoracic and mediastinal disorders**	**4 (9.5)**	**17 (12.3)**	**1 (2.2)**						**22**
Dyspnoea		7 (5.1)							7
Respiratory failure	1 (2.4)	6 (4.3)							7
**Blood and lymphatic system disorders**	**1 (2.4)**	**7 (5.1)**	**1 (2.2)**	**4 (33.3)**					**13**
Anaemia	1 (2.4)	2 (1.4)	1 (2.2)	1 (8.3)					5
**Metabolism and nutrition disorders**	**1 (2.4)**	**6 (4.3)**	**5 (10.9)**						**12**
Decreased appetite	1 (2.4)	6 (4.3)							7
**Eye disorders**	**4 (9.5)**	**3 (2.2)**					**3 (18.8)**		**10**
Eye oedema							2 (12.5)		2
Growth of eyelashes		2 (1.4)							2
Ocular hyperaemia	1 (2.4)						1 (6.3)		2
**Cardiac disorders**		**2 (1.4)**				**2 (66.7)**	**5 (31.3)**		**9**
Bradycardia						2 (66.7)	3 (18.8)		5
**Reproductive system and breast disorders**					**1 (100)**				**1**
Peyronie’s disease					1 (100)				1

Bold type intended as System Organ Classes. ADR, adverse drug reaction; AFT, afatinib; ALEC, alectinib; ALKi, anaplastic lymphoma kinase inhibitors; CER, ceritinib; CRIZ, crizotinib; EGFRi, epidermal growth factor receptor inhibitors; ERL, erlotinib; GEF, gefitinib; NSCLC, non-small cell lung cancer; NTB, nintedanib; OSI, osimertinib; TKIs, tyrosine kinase inhibitors; VEGFi, vascular endothelial growth factor inhibitor; RNF, Rete Nazionale di Farmacovigilanza.

**Table 5:** Detailed description of Sicilian SADR reports related to TKIs for NSCLC.

Case	Type of seriousness	Age (years)	Sex	Suspected drug(s) (Therapeutic indication)	Concomitant drug(s) (Therapeutic indication)	ADR(s)	TTO (days)	Outcome	Causality assessment
1	Hospitalization	74	M	ERL (NSCLC)	Gallopamil, dihydrocodeine	Rash	4	NA	Possible
2	Persistent or significant disability	76	F	ERL (NSCLC)	Acetylsalicylic acid, dexamethasone, omeprazole, lormetazepam	Skin ulcer	110	Fully recovered	Probable
3	Hospitalization	44	F	ERL (NSCLC)	–	Epidermolysis	916	Not yet recovered	Probable
4	Hospitalization	54	M	ERL (NSCLC)	–	Rash	45	Improved	Possible
5	Life-threatening	66	M	ERL (NSCLC)	–	Dyspnoea	146	Recovered with sequelae	Probable
6	Hospitalization	51	F	ERL (NSCLC)	Dexamethasone, furosemide, aciclovir, lansoprazole, enoxaparin	Hemoptysis, cough	132	Improved	Possible
7	Life-threatening	67	M	ERL (NSCLC)	–	Respiratory failure	5	Improved	Probable
8	Death	63	M	GEF (NSCLC)	–	Epilepsy, cerebral ischemia	35	Death after 49 days (ADR-related)	Possible
9	Death	57	M	ERL (NSCLC)	–	Respiratory failure, cardiogenic shock	30	Death after 7 days (unknown cause)	Possible
10	Hospitalization	70	M	ERL (NSCLC)	–	Respiratory failure, hyperpyrexia	342	Fully recovered	Possible
11	Hospitalization	63	F	GEF (NSCLC)	–	Cerebral haemorrhage	64	NA	Possible
12	Death	37	M	ERL (NSCLC III stage)	–	Interstitial lung disease	71	Death after 8 days (unknown cause)	Possible
13	Hospitalization	75	M	ERL (NSCLC)	Lansoprazole, amiodarone, clonidine	Ileus paralytic, hyperpyrexia, tachyarrhythmia	41	NA	Possible
14	Hospitalization	48	F	ERL (NSCLC)	Dexamethasone (dyspnoea), paracetamol (pain)	Neutropenia	6	Fully recovered	Possible
15	Hospitalization	68	M	GEF (NSCLC)	–	Diarrhea, asthenia, dehydration, weight decreased	216	Improved	Probable
16	Life-threatening	65	F	GEF (NSCLC)	–	Pulmonary embolism, renal infarct, hyperamylasemia	63	NA	Possible
17	Hospitalization	63	M	ERL (NSCLC)	–	Dyspnoea, hypertransaminasemia	33	Improved	Probable
18	Hospitalization	58	F	GEF (NSCLC)	–	Hypertransaminasemia	0	Improved	Probable
19	Hospitalization	64	F	GEF (NSCLC)	–	Coma, cellulitis, renal failure, hepatic failure	NA	Improved	Probable
20	Hospitalization	67	M	AFT (NSCLC)	–	Rash, acute myeloid leukaemia	278	Not yet recovered	Possible
21	Hospitalization	74	M	ERL (NSCLC)	–	Respiratory failure	51	Not yet recovered	Possible
22	Hospitalization	81	M	GEF (NSCLC IV stage)	–	Diarrhea	7	NA	Possible
23	Hospitalization	65	M	ERL (NSCLC)	–	Respiratory failure, pneumonia	52	NA	Probable
24	Hospitalization	72	M	ERL (NSCLC)	–	Pneumonia, neutropenia, leukopenia	24	Death after 26 days (unknown cause)	Possible
25	Hospitalization	61	M	CRIZ (NSCLC)	–	Cardiac failure	42	Improved	Probable
26	Death	55	F	OSI (NSCLC)	–	Rash, diarrhea, neutropenia, nail and nail bed conditions, neoplasm progression, general physical health deterioration	NA	Death (unknown cause)	Possible
27	Death	48	M	OSI (NSCLC IV stage)	–	Sudden death	NA	Death (unknown cause)	Possible
28	Hospitalization	65	F	ERL (NSCLC)	–	Leukopenia, pancytopenia, acute promyelocytic leukaemia	1175	Not yet recovered	Probable
29	Hospitalization	53	M	NTB + docetaxel (NSCLC IV stage)	Acetylsalicylic acid + bisoprolol (cardiac disorders), atorvastatin + omega-3-triglycerides (dyslipidemia), levetiracetam (epilepsy)	Gastrointestinal perforation	NA	Not yet recovered	Possible
30	Hospitalization	70	M	AFT (NSCLC)	–	Respiratory failure, pyrexia, interstitial lung disease	504	Improved	Probable
31	Hospitalization	62	F	CRIZ (NSCLC)	Levothyroxine sodium, furosemide, pantoprazole, and desloratadine	Hypertransaminasemia, pericardial effusion	NA	NA	Possible
32	Hospitalization	69	M	CRIZ + pembrolizumab (NSCLC IV stage)	Denosumab (NSCLC IV stage)	Hypertransaminasemia, rash, hepatitis	65	Improved	Highly probable
33	Hospitalization	58	F	OSI (NSCLC III stage)	–	Thrombotic stroke	80	NA	Possible

SADR, serious adverse drug reaction; CRIZ, crizotinib; ERL, erlotinib; GEF, gefitinib; NA, not avilable; NSCLC, non-small cell lung cancer; NTB, nintedanib; OSI, osimertinib; TKIs, tyrosine kinase inhibitors; TTO, time to onset.

## Discussion

The introduction of several TKIs for the treatment of NSCLC has radically changed the management of these patients. Improvements in the identification of molecular subgroups of advanced NSCLC patients led to an increase in the number of molecular analyses requested in this setting (19, 20). However, the use of TKIs is not free from the onset of AEs, even serious ones. In this study, ADRs related to the use of TKIs for the treatment of NSCLC were identified both nationally in the RAM system database and regionally *via* the SRS database. The different timing of approval and the clinical use have been certainly influenced the distribution of ADR reports over the years in Italy and Sicily (21–23). The higher incidence of drug-related ADRs in a specific time frame is linked to the sequential approval of some TKIs, including the newest ones, such as OSI, which replaced the older generation ones, including ERL. Another example is LORL, which arrived in Sicily very recently and unfortunately late compared to other regions (no reports on LORL in Sicily). Moreover, the higher number of reports related to ERL, AFT, and OSI, after their first authorization for NSCLC, is probably due to the inclusion in the AIFA monitoring registers platform; the monitoring was concluded for ERL in 2018, while the monitoring for AFT and OSI was started in 2014 and 2017, respectively, and is still ongoing (24a). Furthermore, some active pharmacovigilance projects were conducted during the years to evaluate the use of biologic drugs in oncologic patients and, in particular, one with a focus on the use of TKIs in NSCLC performed in Campania and Lazio regions through “Pharmacovigilance Funds 2012-2013-2014” had an impact on ADR distributions (25).

Although most ADRs were not serious, SADRs were reported for NTB, LORL, OSI, and ALEC. NTB was associated with a higher incidence of grade 3 ADRs (26). In a recent study, LORL was related to a higher onset of grade 3-4 ADRs than ALEC (27) while, OSI, and ALEC were primarily related to mild-moderate in severity and had a higher tolerability profile also when compared to other TKIs (27, 28). Focusing on gender and age groups, females and the age group >65 years were the most reported as shown in a recent study (29). Moreover, the TTO was similar to those reported in other studies where EGFRi had a median TTO of 28 days especially for gastrointestinal and skin disorders (30), ALKi had a median TTO from 14 to 85 days depending on the type of ADR (31, 32), while NTB had a median TTO of 13.5 days for liver injury (33).

The pathophysiology of TKI-induced skin toxicity is still unclear, but EGFRi block signaling pathways, preventing keratinocytes from maturing properly and impairing migration to the outer stratum corneum. This results in a thinning of the outermost layers of the epidermis and corneal layers, resulting in a loss of the skin’s protective barrier function, increasing sensitivity to UV damage and leading to inflammatory cell recruitment and cutaneous injury. Skin toxicity occurs mainly in areas with high sebaceous content, eccrine glands and other areas with a high expression of EGFR such as the basal layer of the dermis and the pilosebaceous follicle (34, 35). Regarding each single type of ADR, a higher incidence of rash was shown for ERL, GEF, and AFT (29, 36, 37). Moreover, Sicilian data showed a higher onset of xerosis with ERL attributable to an abnormal differentiation of keratinocytes, which consequently leads to a disturbed stratum corneum and loss of water retention function epidermis (35, 38). This finding was confirmed by the 271 cases of dry skin reported in Eudravigilance (39).

A greater occurrence of gastrointestinal reactions, especially diarrhea, was reported for NTB and AFT. Gastrointestinal diseases are common in many oral TKIs acting on EGFR and VEGF (29, 40). Indeed, gastrointestinal disorders were the most reported ADRs for AFT and NTB in the Eudravigilance database (*n*=1,814 and *n*=3,538, respectively) (39). Considering that EGFR is expressed by gastrointestinal epithelial cells, the inhibition of EGFR signaling leads to reduced growth and healing of the intestinal epithelium that induced to mucosal atrophy (41). Moreover, the onset of diarrhea may be caused by excess chloride secretion caused by dysregulated EGFR signaling to downstream pathways and channels (42, 43).

According with other studies, ERL and AFT were mainly associated with the onset of infections such as folliculitis (44, 45), the latter probably attributable to the implication of EGFR signaling pathway in hair cycle regulation and the maintenance of normal hair follicles. TKIs may suppress the progression from the anagen to the telogen phase and lead to the inflammation (29, 46). Folliculitis was one of the most reported infections in Eudravigilance with 109 cases for ERL and 26 cases for AFT (39).

Another important ADR was hypertransaminasemia especially with CER, ALEC, CRIZ, NTB, and GEF. Hepatotoxicity was one of the common ADRs of TKIs, including CRIZ, CER, and NTB (12, 47, 48), while ALEC showed a lower incidence (49). GEF-induced hepatotoxicity seems to be related to an immuno-allergic mechanism and to a dose-dependent cellular toxicity (50) but also it may be associated with polymorphisms in metabolic enzymes, including CYP2D6 (51). Concerning neutropenia, a large number of ADRs was related to NTB probably due to its association with docetaxel (52, 53). Sicilian data also showed a higher onset of decreased appetite with ERL (54); loss of appetite, especially when accompanied by severe and persistent diarrhea, nausea and vomiting may require interruption of the treatment (21). The decreased appetite was the most reported metabolism disorders in the Eudravigilance database in accordance with our result (39). A higher number of reports related to bradycardia was reported for CER. Bradycardia was shown in 3% of patients treated with CER (55). Furthermore, 15 cases were reported in Eudravigilance database (39). Both ALK and ROS1 inhibitors were associated with a higher risk of conduction disease and QT prolongation than other TKIs (56). It is important to monitor the heart rate and the blood pressure to prevent cardiac ADRs (57). In addition, the use of CER in combination with other agents known to cause bradycardia, including beta blockers, clonidine, and digoxin, should be avoided where possible (58).

The case-by-case assessment of serious Sicilian ADR reports showed five deaths related to OSI, ERL, and GEF. Fatal cases mainly involved respiratory failure and interstitial lung disease probably due to the progression of neoplasm and IV staging. A meta-analysis pointed out that patients affected by NSCLC and treated with EGFR-TKIs had a rare incidence of fatal toxic effects although the respiratory system was the most frequently involved (59). Interstitial lung disease is considered to be the most serious and fatal TKI-related ADR because the inhibition of EGFR signaling may damage the repair capability of lung cells and worsen pulmonary injury resulting in death (29). Preventive and therapeutic measures of some comorbid conditions and the neoplasm progression may further improve the outcome in this patient population. For this reason, respiratory ADRs have to be managed abruptly and further research should be undertaken to minimize fatal toxic effects in patients treated with TKIs.

### Strengths and limitations

This is the first European study focusing on ADRs related to the use of TKIs approved for the treatment of NSCLC based on a spontaneous reporting database. Spontaneous reporting System (SRS) is one of the most commonly used pharmacovigilance methods, particularly useful for identifying newly approved drug-related ADRs undetected during pre-marketing studies when the drug is administered to a relatively small number of selected patients and under controlled conditions (60–62). Information coming from multiple ICSRs are a fundamental tool to identify potential safety signals. The use of SRS data has several limitations, including underreporting of suspected ADRs, selective reporting, lack of denominator data (i.e., the total number of patients who have undergone treatment with TKIs), which prevent measuring the absolute risk of suspected ADRs; moreover, SRS relies entirely on individuals’ motivation to report suspected ADRs to a local or national pharmacovigilance center (63). TKIs are mainly used in the advanced stage of NSCLC, in case of relapses or as second-line therapy after treatment with conventional chemotherapy. Consequently, it cannot be excluded that some of the reported ADRs, including cases of death, may be due to the neoplasm progression, to the neoplasm staging, to the onset of delayed ADRs or to some comorbidities in cancer patients treated with TKIs (29). In addition, Naranjo’s algorithm for causality assessment also has limitations such as lack of sensitivity because many responses are classified as unknown in the absence of data that does not always allow the evaluation of possible alternative causes. It also has a serious disadvantage in verifying or invalidating the causality and, at the same time, is not able to provide accurate information on the quantitative extent of the probability assessment (64). Furthermore, the MedDRA PT classification has some limitations, in particular to identify the correct PT that can be reported with different PT synonyms for the same clinical condition, which leads to the false representation of reported ADR (65). A detailed description of ADR reports is available only for Sicilian data collected into the Italian SRS database and recently approved drugs, including BRG and LORL, were not detected in the Sicilian database. Despite these limitations, it is known that SRS contributes to the characterization of safety profiles, which is particularly important for preventing some ADRs related to the use of TKIs in the treatment of NSCLC.

### Conclusions

The importance of the SRS database for the identification of TKI-induced ADRs for the treatment of NSCLC was shown in the present study. The analysis of spontaneous ADR reports of TKIs confirmed, in general, well-known risks associated to these drugs in accordance with the frequency of AEs described in each Summary of Product Characteristic, which often include skin, gastrointestinal, general, liver, and respiratory diseases as well as infections. All TKIs are still better tolerated than chemotherapy and generally have better therapeutic results. Our findings were a fundamental tool to help the oncologists in the management of ADRs in clinical practice, especially for some kind of toxicity. Furthermore, more attention should be paid to the occurrence of serious ADRs including respiratory failure, interstitial lung disease, and cardiogenic shock as they are the ones inducing to death even in young patients. More analyses should be conducted to get further information on the safety profile of TKIs considering their effectiveness in treating different types of cancer, but also for the onset of ADRs that can reduce therapy compliance and should be recognized and managed as soon as possible. Close collaboration between oncologists and pharmacologists is required to early identify ADRs, make patients aware of the SRS and thus incentivize reporting to reduce risks appropriately.

## Data availability statement

The datasets presented in this study can be found in online repositories. The names of the repository/repositories and accession number(s) can be found below: The datasets generated for this study will not be made publicly available. National dataset in aggregated form is available online, while the access to the regional in single, non-aggregated dataset requires the approval of the Italian Medicines Agency.

## Author contributions

All authors listed have sufficiently contributed to the entire content of the manuscript and have given their consent for publication. Project coordination: ES. Acquisition of data: PMC and ES. Analysis and interpretation of data: MAB, EES, GC, and VS. Clinical evaluation of data: NS and ES. Wrote the paper: MAB, EES, and PMC. Critical revision: PMC, TF, MS, NS, and ES. Final approval of the version to be published: MAB, EES, GC, VS, PMC, TF, MS, NS, and ES. All authors contributed to the article and approved the submitted version.

## Conflict of interest

The authors declare that the research was conducted in the absence of any commercial or financial relationships that could be construed as a potential conflict of interest.

## Publisher’s note

All claims expressed in this article are solely those of the authors and do not necessarily represent those of their affiliated organizations, or those of the publisher, the editors and the reviewers. Any product that may be evaluated in this article, or claim that may be made by its manufacturer, is not guaranteed or endorsed by the publisher.
