# Supplementary material for: Safety profile of tyrosine kinase inhibitors used in non-small-cell lung cancer: An analysis from the Italian pharmacovigilance database
Source: Front Oncol. 2022 Nov 23;12:1005626. doi: 10.3389/fonc.2022.1005626 (PMC9727240; doi:10.3389/fonc.2022.1005626)
Supplement: Supplementary file 1 [file DataSheet_1.pdf]

Supplementary Table 1. ADR groupings by clustering the synonymous PTs of the same clinical condition under one term

Clinical condition	Preferred Term (PT)
Liver injury	Liver injury
	Drug-induced liver injury
	Hepatotoxicity
	Liver disorder
	Hepatic function abnormal
	Liver function test abnormal
Lymphopenia	Lymphocyte count decreased
	Lymphopenia
Thrombocytopenia	Platelet count decreased
	Thrombocytopenia
Neutropenia	Neutrophil count decreased
	Neutropenia
Leukopenia	Leukopenia
	White blood cell count decreased
Renal failure	Renal failure
	Acute kidney injury
	Renal impairment
Vomiting	Vomiting
	Retching
Hypertransaminasemia	Transaminases increased
	Hypertransaminasemia
	Aspartate aminotransferase increased
	Alanine aminotransferase increased
Rash	Skin reaction
	Skin lesion
	Skin erosion
	Rash
	Rash erythematous
	Erythema
	Drug eruption
	Skin exfoliation
	Toxic skin eruption
	Eczema
Abdominal pain	Abdominal pain
	Abdominal pain upper
	Abdominal tenderness
Bradycardia	Heart rate decreased
	Sinus bradycardia
	Bradycardia
Cardiac fibrillation	Atrial fibrillation
	Ventricular fibrillation
Hyperbilirubinaemia	Blood bilirubin increased
	Hyperbilirubinaemia
Asthenia	Asthenia
	Fatigue
Dyspnea	Dyspnea
	Dyspnea exertional
	Nocturnal dyspnoea
Hypercreatininaemia	Blood creatinine increased
	Hypercreatininaemia
Visual impairment	Visual impairment
	Vision blurred
Gastroenteritis	Gastroenteritis
	Enteritis
Viral infection	Cytomegalovirus infection

	Coronavirus infection
	BK virus infection
	Coronavirus test positive
Hepatic failure	Hepatic failure
	Acute hepatic failure
Respiratory failure	Respiratory failure
	Acute respiratory failure
	Respiratory distress
Dyslipidaemia	Dyslipidaemia
	Hypercholesterolaemia
	Hypertriglyceridaemia
	Blood triglycerides increased
	Blood cholesterol increased
Pneumonia	Pneumonia
	Pneumonia pseudomonal
Hallucination	Hallucination
	Hallucinations, mixed
Thrombocytosis	Thrombocytosis
	Platelet count increased
Neoplasm progression	Disease progression
	Neoplasm progression
	Condition aggravated
	Malignant neoplasm progression
	Non-small cell lung cancer
	Adenocarcinoma
Gastrointestinal perforation	Intestinal perforation
	Large intestine perforation
	Gastrointestinal perforation
	Jejunal perforation
	Duodenal ulcer perforation
Hyperamylasaemia	Amylase increased
	Hyperamylasaemia
Hypercreatinaemia	Hypercreatinaemia
	Blood creatine increased
Multiple organ dysfunction syndrome	Multi-organ disorder
	Multiple organ dysfunction syndrome
Off label use	Product use in unapproved indication
	Off label use
Hypersensitivity	Hypersensitivity
	Drug hypersensitivity
Oral pain	Oral pain
	Oropharyngeal pain
Eye edema	Eyelid edema
	Eye edema
Ocular hyperaemia	Conjunctival hyperemia
	Ocular hyperaemia
Abnormal loss of weight	Abnormal loss of weight
	Weight decreased
Nail and nail bed conditions	Nail toxicity
	Nail disorder
	Ingrowing nail
	Nail discolouration
	Nail dystrophy
	Nail infection
	Onychoclasis
	Onycholysis
	Onychomalacia
	Onychomycosis
	Paronychia

Skin toxicity	Skin disorder
	Skin toxicity
Cardiac flutter	Cardiac flutter
	Atrial flutter
Dermatitis	Dermatitis
	Dermatitis acneiform
	Dermatitis allergic
	Dermatitis exfoliative
	Dermatitis psoriasiform
	Seborrhoeic dermatitis
Pemphigoid	Dermatitis bullous
	Pemphigoid
Hyperglycaemia	Hyperglycaemia
	Blood glucose increased
Epilepsy	Petit mal epilepsy
	Epilepsy
Hyperbilirubinaemia	Blood bilirubin increased
	Hyperbilirubinaemia
Anaemia	Blood loss anaemia
	Anaemia
Hypertension	Hypertension
	Blood pressure increased
Hyperuricaemia	Blood uric acid increased
	Hyperuricaemia
Hyperkalaemia	Blood potassium decreased
	Hyperkalaemia
Gastrointestinal ulcer	Duodenal ulcer
	Gastric ulcer
	Gastroduodenal ulcer
Gastrointestinal disorder	Gastrointestinal motility disorder
	Gastrointestinal disorder
Vomiting	Vomiting projectile
	Vomiting
Pruritus	Rash pruritic
	Pruritus
	Prurigo
